# Tunable high-quality-factor absorption in a graphene monolayer based on quasi-bound states in the continuum

**DOI:** 10.3762/bjnano.13.59

**Published:** 2022-07-19

**Authors:** Jun Wu, Yasong Sun, Feng Wu, Biyuan Wu, Xiaohu Wu

**Affiliations:** 1 College of Electrical Engineering, Anhui Polytechnic University, Wuhu, 241000, China; 2 Basic Research Center, School of Power and Energy, Northwestern Polytechnical University, Xi’an 710064, Shaanxi, China; 3 Center of Computational Physics and Energy Science, Yangtze River Delta Research Institute of NPU, Northwestern Polytechnical University, Taicang 215400, Jiangsu, China; 4 School of Optoelectronic Engineering, Guangdong Polytechnic Normal University, Guangzhou 510665, China; 5 Shandong Institute of Advanced Technology, Jinan 250100, China

**Keywords:** bound states in the continuum, graphene, gratings, selective absorption

## Abstract

A tunable graphene absorber, composed of a graphene monolayer and a substrate spaced by a subwavelength dielectric grating, is proposed and investigated. Strong light absorption in the graphene monolayer is achieved due to the formation of embedded optical quasi-bound states in the continuum in the subwavelength dielectric grating. The physical origin of the absorption with high quality factor is examined by investigating the electromagnetic field distributions. Interestingly, we found that the proposed absorber possesses high spatial directivity and performs similar to an antenna, which can also be utilized as a thermal emitter. Besides, the spectral position of the absorption peak can not only be adjusted by changing the geometrical parameters of dielectric grating, but it is also tunable by a small change in the Fermi level of the graphene sheet. This novel scheme to tune the absorption of graphene may find potential applications for the realization of ultrasensitive biosensors, photodetectors, and narrow-band filters.

## Introduction

Absorbers possess a wide range of applications, including radar stealth, infrared detectors, thermophotovoltaic cells, and thermal emitters. According to their spectral bandwidths, the absorbers can be classified as broad-band absorbers and narrow-band absorbers [1–4]. In general, broad-band absorbers [5–8] are used for electromagnetic cloaking and solar energy conversion, while narrow-band absorbers [9–12] have great potential in sensing and monochromatic light detecting. An example is the application of narrow-band absorbers in refractive index sensing [13]. When the absorbers are surrounded by gas or liquid, the resonance wavelength will shift as the background refractive index changes [14]. Narrow-band absorbers have attracted attention in practical applications due to the absorption with high quality factor (Q-factor), which is beneficial to improve the sensing performance. Up to now, many strategies for improving the Q-factor have been successfully proposed, such as dielectric resonant [15], all-metal [14,16] and metal–dielectric–metal (MDM) configurations [17–18]. Recently, as a type of particular localized states, optical bound states in the continuum (BICs) [19–24] have also been demonstrated to enable perfect light confinement and giant field enhancement [25–29]. Hence, quasi-BICs can be utilized to design narrow-band absorbers with high Q-factor.

Tunable absorption is interesting regarding many potential applications. There are generally two ways to achieve tunable absorption. One is to change the structural parameters and the other is to add tunable materials, such as phase change materials, graphene, or liquid crystals. Among them, graphene has attracted much attention in optics and optoelectronics [30–34]. As a single layer of carbon atoms arranged in a honeycomb structure, graphene supports much stronger binding of surface plasmon polaritons (SPPs) with less loss, which leads to a longer propagation distance compared with traditional metal SPPs [35]. In addition, its conductivity can be dynamically controlled by chemical doping or electrostatic fields owing to the linear dispersion of the Dirac fermions [36]. These features proposed for graphene enable novel active devices, including modulators [37], perfect absorbers [38–39], imaging devices [40], detectors [41], waveguides [42–43], polarizers [44], and electromagnetic chirality devices [45]. The strength of interaction between graphene and incident light plays a key role in these applications. Unfortunately, it is extremely weak owing to the single-atom thickness of graphene monolayers, which severely limits the performance of graphene devices. Various approaches based on different physical mechanisms have been proposed to enhance the absorption in graphene monolayers, such as coherent perfect absorption effect [46], critical coupling effect [47], guided mode resonance effect [48], metal Tamm plasmon polaritons effect [49], and graphene Tamm surface plasmons effect [50]. In addition, the quasi-BICs mentioned previously also can be employed to enhance the absorption in graphene monolayers [51–54]. However, in the above works, the quasi-BICs are quite sensitive to the geometric parameters, which limit their practical applications. Besides, in [51–53], the proposed structures are metasurfaces, which makes the fabrication quite difficult.

In this paper, motivated by the investigation in [28], a tunable graphene absorber, which consists of a graphene monolayer on a dielectric grating backed with a substrate, is designed and investigated. The paper is arranged as follows: First, we present the structure of the absorber and give the corresponding geometric parameters. Second, the absorption properties are calculated and the electromagnetic field distributions at the resonant wavelength are investigated to disclose the physical origin of enhanced absorption. Next, the spatial directivity is discussed so as to find its potential applications as thermal emitter with high directivity. Finally, the properties of the active absorber, including tunability with different geometric parameters and different Fermi levels are investigated.

## Results

**Figure 1:**
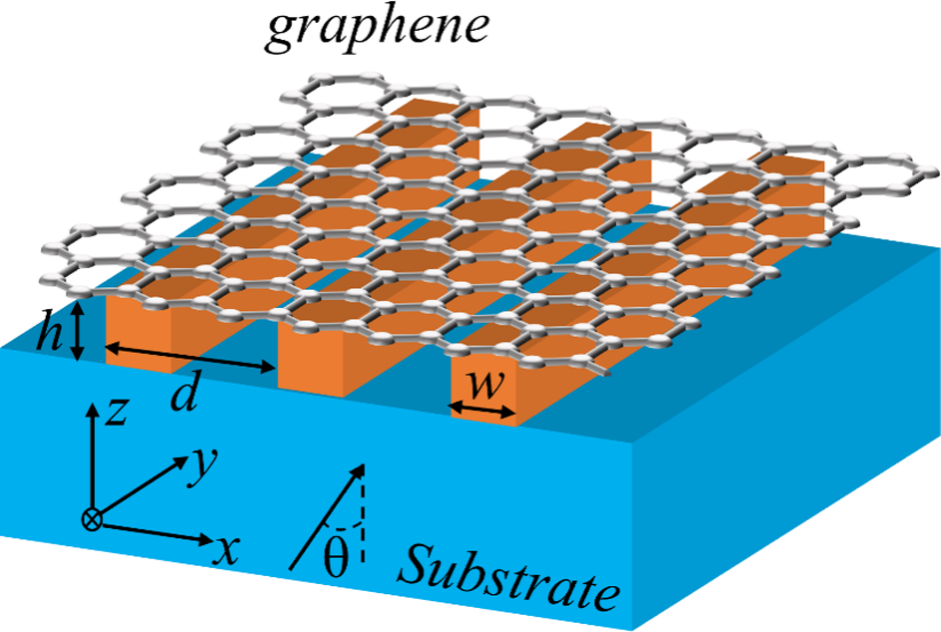
Schematic of the graphene absorber consisting of a graphene monolayer and a substrate separated by a dielectric grating.

Figure 1 gives a schematic view of the proposed absorber. A graphene monolayer is placed on a one-dimensional dielectric grating, under which a dielectric substrate is used to support the device. The dielectric grating is defined by the period *d*, the width *w*, and the thickness *h*. The refractive indices of grating and substrate are *n*_h_ and *n*_s_, respectively. TM polarized (the magnetic field is along the direction of the *y*-axis) monochromatic plane waves are incident from the substrate at an angle θ. In our simulation, we set *d* = 3.3 μm, *w* = 2.31 μm, *h* = 3.5 μm, *n*_h_ = 3.48, *n*_s_ = 1.45, and θ = 0.1°.

The surface conductivity of graphene has intraband and interband contributions and is described by:


[1] $$\sigma \left(\omega \right)=\sigma_{\text{intra}}\left(\omega \right)+\sigma_{\text{inter}}\left(\omega \right).$$

Here, σ_intra_ and σ_inter_ are the intraband and interband conductivity, respectively. In the mid-infrared wavelength region considered in this work, the Fermi level is greater than half of the photon energy, that is, ℏω < 2*E*_f_. Thus, the intraband contribution will dominate the graphene conductivity as the interband transitions are negligible due to Pauli blocking. Therefore, the conductivity of graphene can be approximately expressed by the Drude-like surface conductivity σ_intra_ as follows [55–56]:


[2] $$\sigma \left(\omega \right)=\frac{e^{2}E_{\text{f}}}{\pi \hbar^{2}}\frac{i}{\omega +i\tau^{-1}},$$

where ℏ is the reduced Planck’s constant, *E*_f_ is the energy of the Fermi level, ω is the angular frequency, *e* is the elementary charge, and τ is the carrier relaxation lifetime.

In our simulation, the permittivity of the graphene monolayer is described by:


[3] $$\varepsilon_{\text{g}}=1+\frac{i\sigma \left(\omega \right)}{\varepsilon_{0}\omega h_{\text{g}}},$$

where ε_0_ is the relative permittivity of vacuum, and *h*_g_ is the thickness of the graphene, which is assumed to be 0.34 nm. Throughout this work, we assume τ = 0.1 ps. The Fermi level is initially considered to be *E*_f_ = 0.3 eV, and its influence on absorption spectra will be analyzed latter.

**Figure 2:**
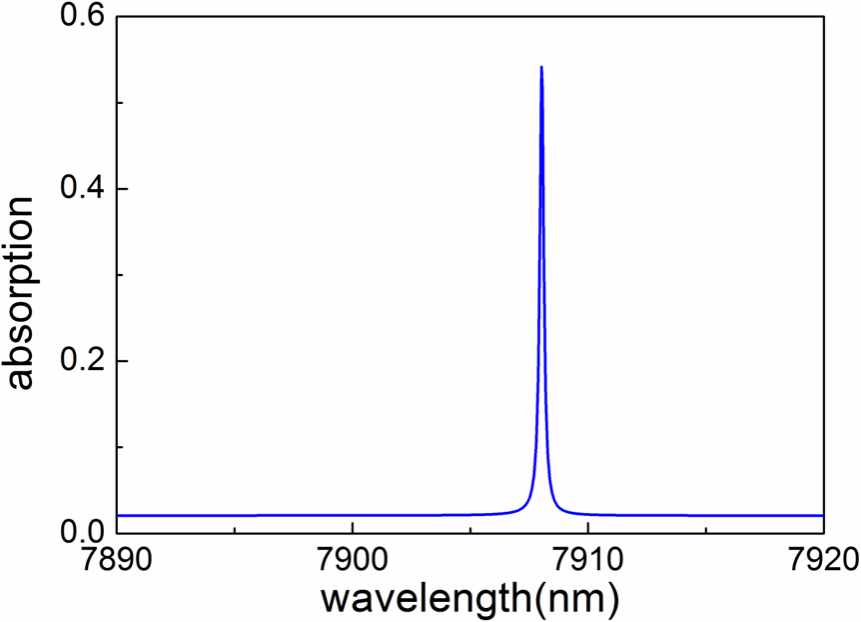
Simulated absorption spectrum for the graphene absorber with operating wavelength at about 7908.03 nm. Here, θ = 0.1°.

Based on the parameters mentioned above, we calculated the absorption spectra shown in Figure 2. The absorption spectra *A*(λ) are obtained from reflection spectra *R*(λ) and transmission spectra *T*(λ) through *A*(λ) = 1 − *R*(λ) − *T*(λ), where, *R*(λ) and *T*(λ) are calculated by employing the rigorous coupled wave analysis (RCWA) method [57–58]. Clearly, a sharp strong resonant absorption peak is observed at a wavelength of 7908.03 nm. The absorption at the resonant wavelength is about 54.13%. The enhanced absorption at this wavelength is attributed to the excitation of quasi-BICs, which will be verified in the following. Moreover, the corresponding Q-factor is about 37657, which yields an ultrasharp absorption profile. Here, the Q-factor is defined by *Q* = λ/Δλ, where λ is the resonant wavelength of the absorption peak and Δλ denotes the full width at half maximum of the peak.

**Figure 3:**
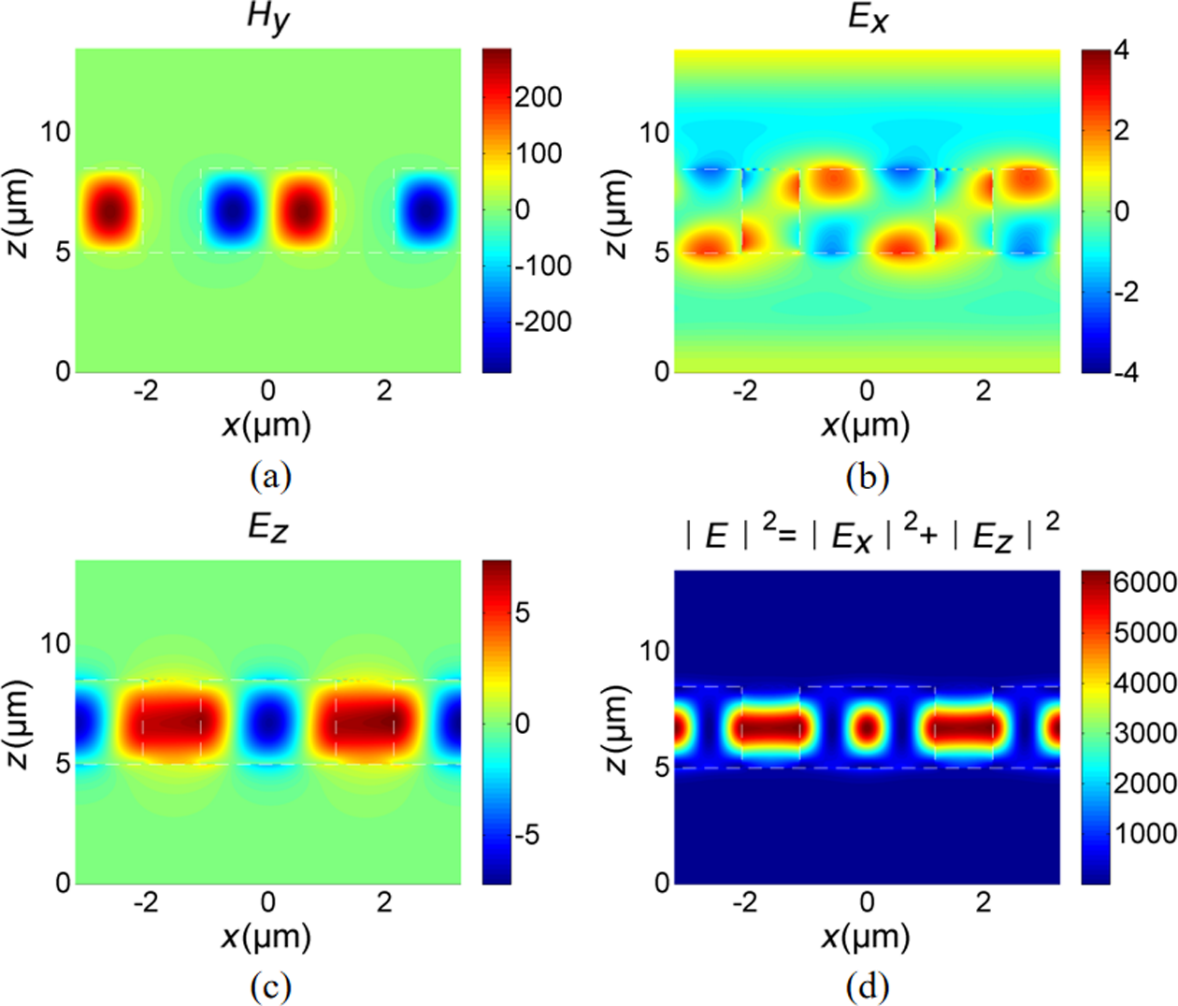
Electromagnetic field distributions: (a) real(*H_y_*), (b) real(*E_x_*), (c) real(*E_z_*), and (d) |*E*|^2^ = |*E_x_*|^2^ + |*E_z_*|^2^. The regions enclosed by the white dash line are the dielectric grating. A graphene monolayer, which cannot be displayed due to its ultrathin thickness, is attached to the dielectric grating. The origin of the *z*-axis is located at a surface 5 μm below the dielectric grating.

To examine the physical mechanism of this phenomenon, we illustrate the distributions of the electromagnetic field at the resonant wavelength in Figure 3. Clearly, when the dielectric grating is illuminated by a TM polarized light under nearly normal incidence, the resonator will generate field distributions similar to an electric dipole [28]. At this point, *H_y_* and *E_x_* are antisymmetric with respect to the *y*–*z* plane, as illustrated in Figure 3a and Figure 3b. Now, radiation in the *z*-axis direction will be forbidden as the symmetry of field inside the grating is mismatched with the external field distributions [28]. At the same time, radiation to the off-*z*-axis direction is also forbidden because only zero-order radiation is permissible for light under normal incidence owing to the subwavelength unit cell of this structure. Therefore, the radiation mode will be confined in the dielectric grating, which results in large electric field intensity enhancement and concentration inside the grating, as presented in Figure 3d. For a nonmagnetic dispersive medium, the time-averaged power loss density is described by [59]: d*P*_loss_/d*V* = 1/2ε_0_ω·Im (ε(ω))|*E*|^2^, where Im(ε) denotes the imaginary part of relative permittivity and *E* is the electric field. Thus, the strong electric intensity enhancement inside the dielectric grating will boost light absorption in the graphene monolayer when it is attached on the grating. The enhanced absorption in the graphene monolayer is attributed to the excitation of optical quasi-BICs of radiation modes.

**Figure 4:**
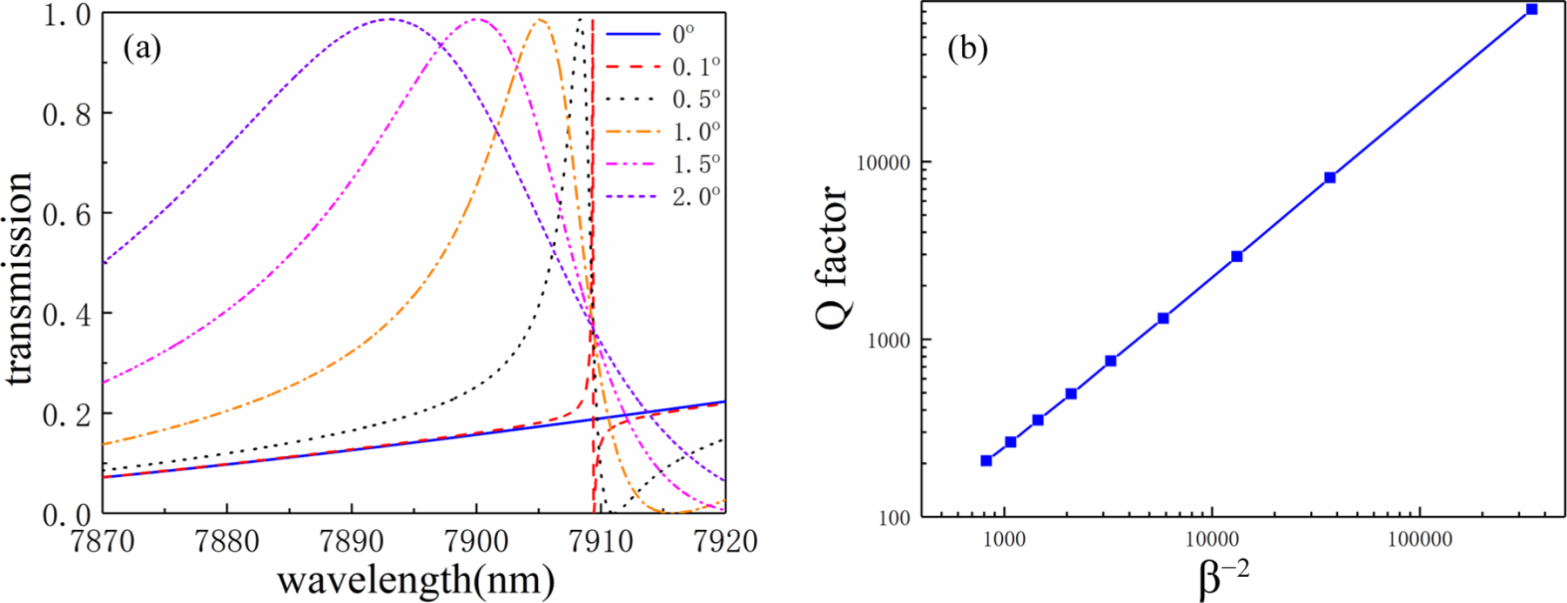
(a) Simulated zero-order transmission spectra of the structures without graphene monolayer for angles of 0°, 0.1°, 0.5°, 1.0°, 1.5°, and 2.0°; (b) dependence of the Q-factor on the inverse square of the asymmetric parameter β^−2^ (log–log scale).

To confirm that the absorption originates from quasi-BICs, we show the simulated zero-order transmission spectra of the structures without graphene monolayer for angles of 0°, 0.1°, 0.5°, 1.0°, 1.5°, and 2.0° in Figure 4a. Clearly, with the successively reduction of the incident angle from 2.0° to 0°, the bandwidth of the Fano resonance peak decreases rapidly. At θ = 0°, the bandwidth has completely vanished, indicating an infinite Q-factor. To further confirm the formation of the BICs, we define an asymmetric parameter β = sin θ and give the dependence of the Q-factor on the inverse square of the asymmetric parameter β^−2^ in Figure 4b (in log–log scale). Here, the Q-factor is calculated by *Q* = λ_peak_/|*λ*_Peak_ − *λ*_dip_| [60]. It is found that, in the log–log scale, the Q-factor almost linearly depends on β^−2^, indicating the formation of BICs in the proposed structure [53].

## Discussion

**Figure 5:**
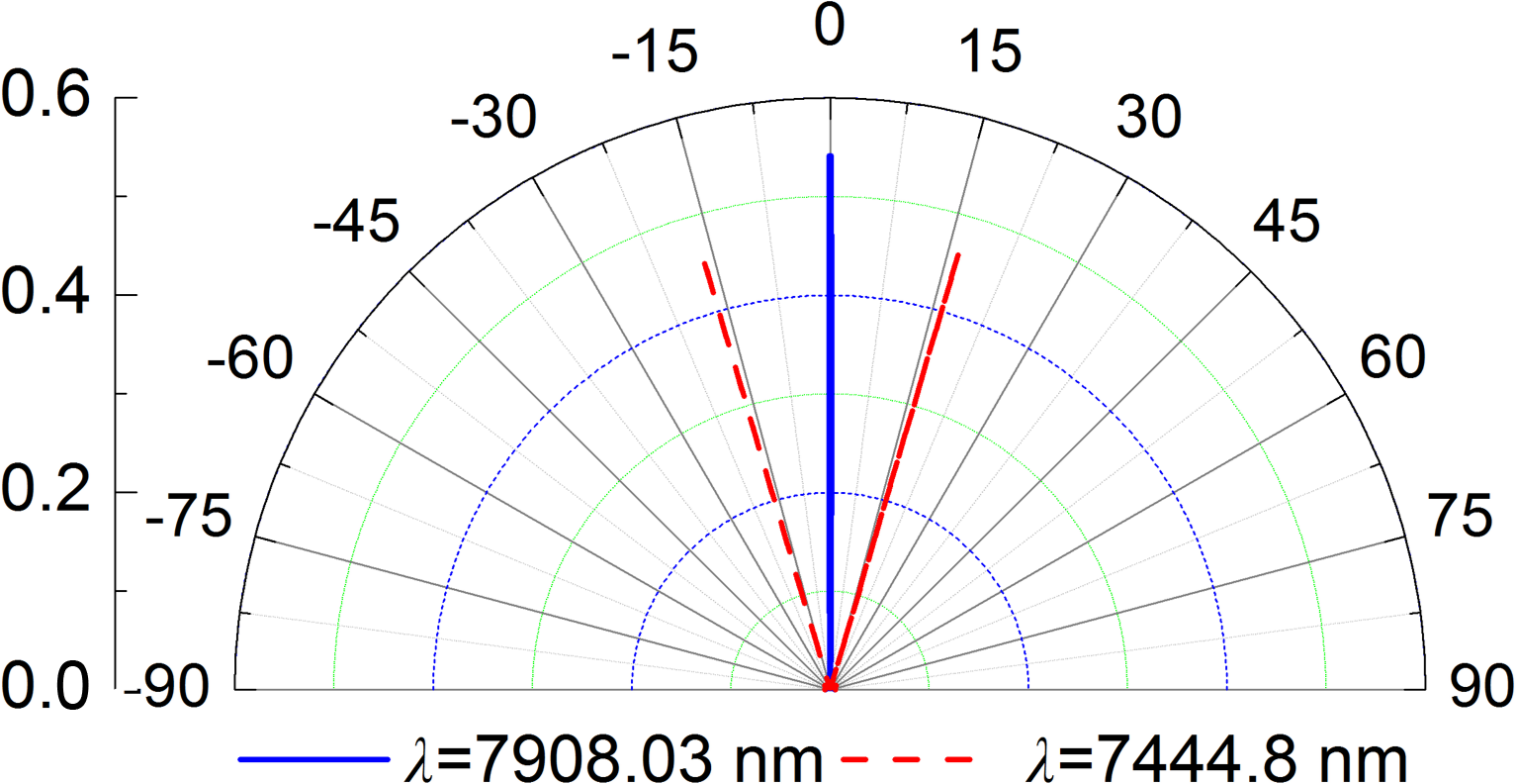
Polar plot of the absorption at wavelengths of *λ*_1_ = 7908.03 nm and *λ*_2_ = 7444.8 nm.

Subsequently, we examine the spatial directivity of the absorber. In Figure 5, we show the polar plot of absorption at wavelengths of λ_1_ = 7908.03 nm and λ_2_ = 7444.8 nm. Obviously, the peak absorptions of λ_1_ and λ_2_ are in the direction of 0° and 16.3°, respectively. In addition, their corresponding angular widths are about 0.22° and 0.086°, respectively, which are ultranarrow angular bandwidths. Therefore, the proposed absorber possesses excellent spatial coherence as the inverse relationship between coherence length and its angular width [61]. Usually, the emissivity is the same as the absorptivity, according to the Kirchhoff's law [61]. The spectral emissivity can be obtained by multiplying the spectral absorptivity with the black-body radiation. Thus, the designed absorber could also be employed to achieve highly directional thermal emission.

**Figure 6:**
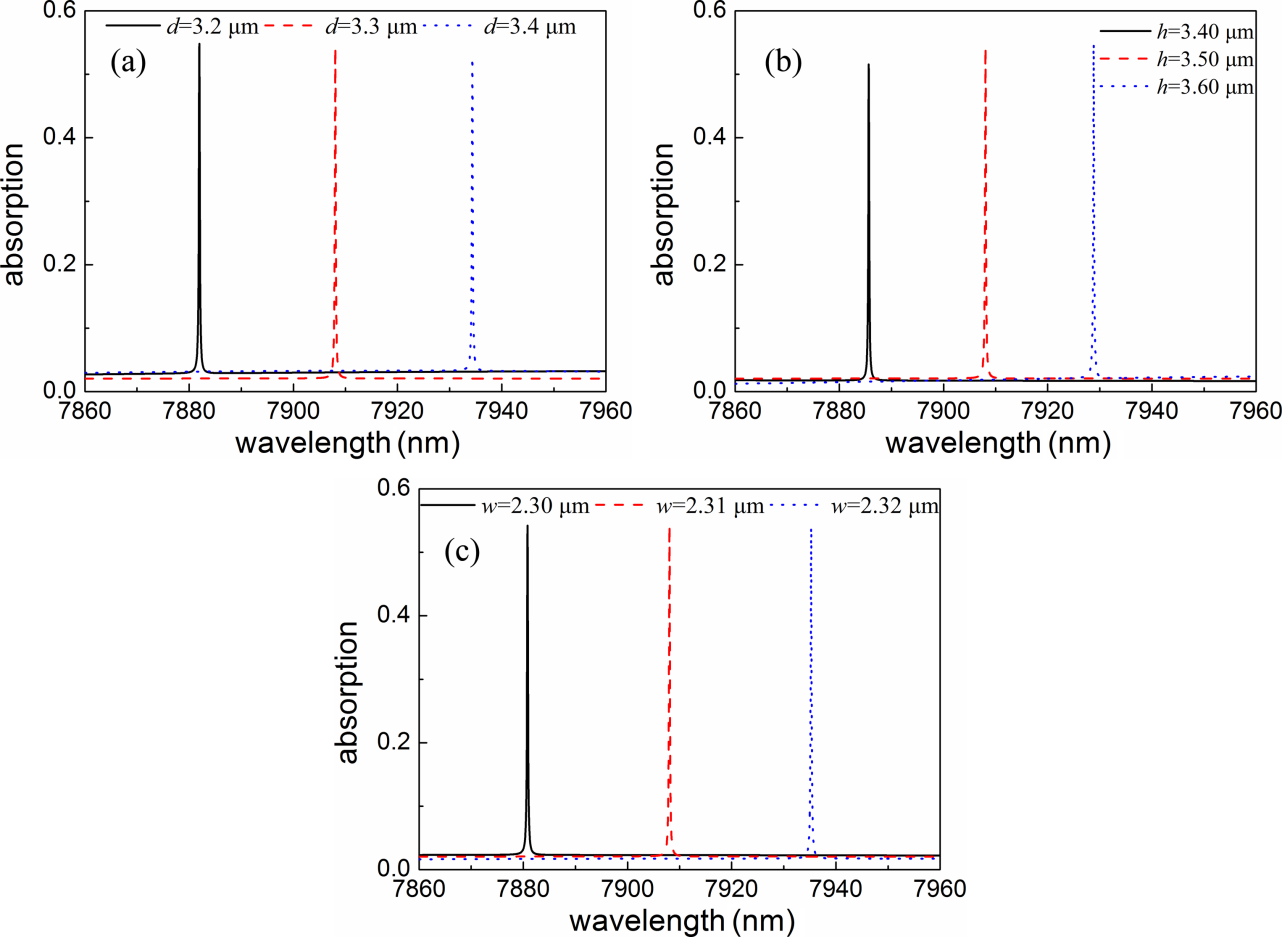
Absorption spectra of the graphene absorbers for different values of (a) *d*, (b) *h* and (c) *w*.

As mentioned above, the absorber has an ultrasharp absorption profile due to the formation of optical quasi-BICs, indicating that the absorption is sensitive to a change of the geometric parameters. Therefore, the geometric tolerance should be precisely controlled inside a certain range during fabrication. We investigate the influence of geometric parameters on the absorption spectra so as to provide a useful guidance for practical fabrication. The results are shown in Figure 6. As shown in Figure 6a, increasing *d* from 3.2 to 3.4 μm results in the shift of the resonant peak to longer wavelengths. This trend is similar to the change of *d* when *h* or *w* are increased. Moreover, the absorption remains almost unchanged with the change of geometric parameters. It is worth noting that the absorption is more sensitive to the change of *w* than to that of *d* and *h*. In general, the ultrasharp absorption can be maintained with a large tolerance regarding the geometric parameters, which is beneficial for real-life fabrication.

**Figure 7:**
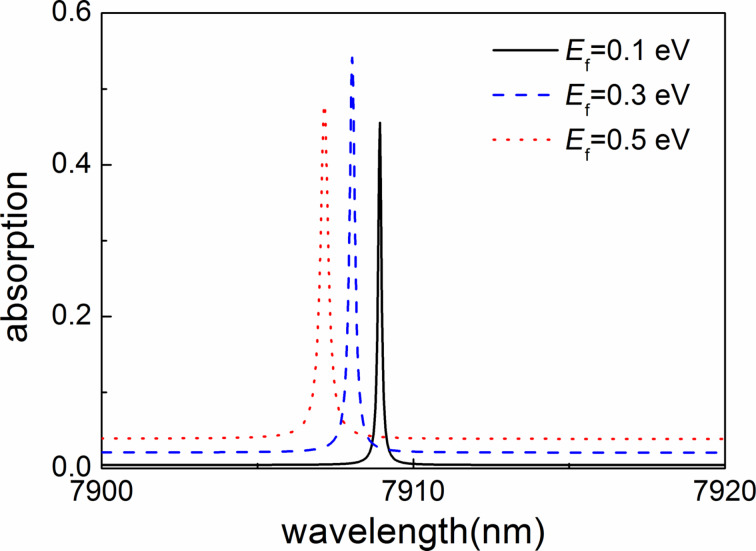
Absorption spectra with different Fermi levels. The geometric parameters of the absorber are the same as before.

As the surface conductivity of the graphene sheet is proportional to the Fermi level (Equation 2), a change of the Fermi level should have direct influence on the graphene absorption. In Figure 7, we show the absorption spectra for different Fermi levels. Obviously, the spectral absorptivity exhibits a blueshift with *E*_f_ increasing from 0.1 to 0.5 eV. In addition, the corresponding resonant absorption increases first and then decreases again. Thus, the ultranarrow absorption could be dynamically controlled through changing the Fermi level without re-designing and re-fabricating the structure, which should be attractive for real-life applications.

Although the theoretical Q-factor of BICs for the dielectric resonant gating is nearly infinite, the practical Q-factor with the fabricated grating only has a large finite value. As clearly shown in Figure 6, the ultrasharp absorption can be maintained with large tolerance regarding the geometric parameters. This means that the high Q-factor of the proposed scheme is robust to the change of the geometric dimensions, which should be beneficial for real-life applications. Only theoretical design and analysis are presented in this work. For real-life applications, we could first fabricate the substrate-supported dielectric grating by means of traditional lithography, and then employ the conventional wet-base transfer method to transfer a CVD-grown graphene monolayer onto the grating structure.

## Conclusion

In summary, an active graphene absorber, consisting of a graphene monolayer and a substrate spaced by a dielectric grating, is proposed and investigated. The absorber exhibits an absolute absorption of more than 50% using a graphene monolayer, which is attributed to the extremely high field enhancement in graphene associated with embedded bound states in the dielectric grating. The electromagnetic field distributions confirm the physical origin of this phenomenon. The proposed absorber has an ultranarrow absorption profile with ultrahigh Q-factor and high spatial directivity, which enables the use as a thermal emitter with high spatial directivity. We also found that the spectral position of the absorption peak can be changed without degrading performance by adjusting the geometrical parameters. This indicates a large geometric tolerance, which is advantageous for fabrication. More importantly, the operating wavelength can be tuned by only a small change in the Fermi level, which is particularly attractive as the absorption properties can be electrically tuned without re-fabricating the whole structure. The results may find potential applications for the realization of high-performance graphene-based electrically tunable active devices including ultrasensitive biosensors, detectors, and perfect filters.
